# The human lncRNA LINC-PINT inhibits tumor cell invasion through a highly conserved sequence element

**DOI:** 10.1186/s13059-017-1331-y

**Published:** 2017-10-27

**Authors:** Oskar Marín-Béjar, Aina M. Mas, Jovanna González, Dannys Martinez, Alejandro Athie, Xabier Morales, Mikel Galduroz, Ivan Raimondi, Elena Grossi, Shuling Guo, Ana Rouzaut, Igor Ulitsky, Maite Huarte

**Affiliations:** 1 Department of Gene Therapy and Regulation of Gene Expression, Center for Applied Medical Research, University of Navarra, Pamplona, 31008 Spain; 2 Institute of Health Research of Navarra (IdiSNA), Pamplona, Spain; 3 Present Address: Laboratory for Molecular Cancer Biology, Department of Oncology, KU Leuven, Leuven, Belgium; 4 Department of Immunology and Immunotherapy, Center for Applied Medical Research, University of Navarra, Pamplona, 31008 Spain; 5 Department of Antisense Drug Discovery and Clinical Development, Ionis Pharmaceuticals, Carlsbad, CA USA; 6 Department of Biological Regulation, Weizmann Institute of Science, Rehovot, 76100 Israel

**Keywords:** LncRNA, Cancer, Cell invasion, Epigenetic regulation, PRC2

## Abstract

**Background:**

It is now obvious that the majority of cellular transcripts do not code for proteins, and a significant subset of them are long non-coding RNAs (lncRNAs). Many lncRNAs show aberrant expression in cancer, and some of them have been linked to cell transformation. However, the underlying mechanisms remain poorly understood and it is unknown how the sequences of lncRNA dictate their function.

**Results:**

Here we characterize the function of the p53-regulated human lncRNA LINC-PINT in cancer. We find that LINC-PINT is downregulated in multiple types of cancer and acts as a tumor suppressor lncRNA by reducing the invasive phenotype of cancer cells. A cross-species analysis identifies a highly conserved sequence element in LINC-PINT that is essential for its function. This sequence mediates a specific interaction with PRC2, necessary for the LINC-PINT-dependent repression of a pro-invasion signature of genes regulated by the transcription factor EGR1.

**Conclusions:**

Our findings support a conserved functional co-dependence between LINC-PINT and PRC2 and lead us to propose a new mechanism where the lncRNA regulates the availability of free PRC2 at the proximity of co-regulated genomic loci.

**Electronic supplementary material:**

The online version of this article (doi:10.1186/s13059-017-1331-y) contains supplementary material, which is available to authorized users.

## Background

Over the last decades, researchers have dedicated great efforts to find the gene alterations that influence the development of cancer. For the most part, these investigations have solely focused on protein-coding genes, while the vast majority of the genome does not code for proteins and most of the mutations associated with disease lie within non-coding regions [1]. Significantly, an important part of the non-coding genome is transcribed to produce non-coding RNAs, and a subset of them are long (>200 nt), capped, and polyadenylated transcripts transcribed by RNA polymerase II, collectively called long non-coding RNAs (lncRNAs) [2].

It is now clear that many lncRNAs can regulate genome function and gene expression [3, 4]. In agreement with this, others and we have observed that alterations in lncRNAs are inherent to cancer and impact several hallmarks of the disease (reviewed in [5–8]). The existence of thousands of lncRNAs taking part in cell regulatory networks has important implications for cancer, forcing us to revise our view of the disease, from its causative origins to treatments. However, still little is known of how lncRNAs contribute to the transformed phenotype of cancer cells. Since the nature of the sequences and the molecular interactions that confer functionality to lncRNAs remain poorly understood, one of the major challenges is to identify the sequence elements that allow lncRNAs to carry out their activities. A puzzling feature of lncRNAs is their relatively low conservation across species. In fact, many human lncRNAs are not present in other organisms, while others, although found in other species, have a limited degree of sequence conservation. These sequences probably contain elements necessary for their activity [9–13].

Among the variety of mechanisms reported, a number of lncRNAs have been proposed to regulate gene expression in coordination with the Polycomb Repressive Complex 2 (PRC2) [14–16]. PRC2 catalyzes the tri-methylation of histone H3 at lysine 27 (H3K27me3), a mark of silent chromatin, and while PRC2 is essential for development, its deregulation leads to cancer progression (reviewed by [17–19]). Multiple lncRNAs have been shown to interact with this chromatin complex, although the significance of these findings is currently under active debate [20–22].

Here we characterize the function of the human lncRNA LINC-PINT in cancer. We found that LINC-PINT acts as tumor suppressor lncRNA that inhibits the migration capacity of cancer cells by repressing an invasion gene signature in a PRC2-dependent manner. Moreover, we show that the functionality of LINC-PINT resides in a highly conserved sequence motif that mediates the interaction with PRC2. We propose that LINC-PINT may function as a DNA decoy that provides PRC2 to active gene promoters for their silencing, a mechanism that could be shared by other PRC2-interacting lncRNAs.

## Results

### LINC-PINT is downregulated in multiple types of cancer

**Fig. 1:**
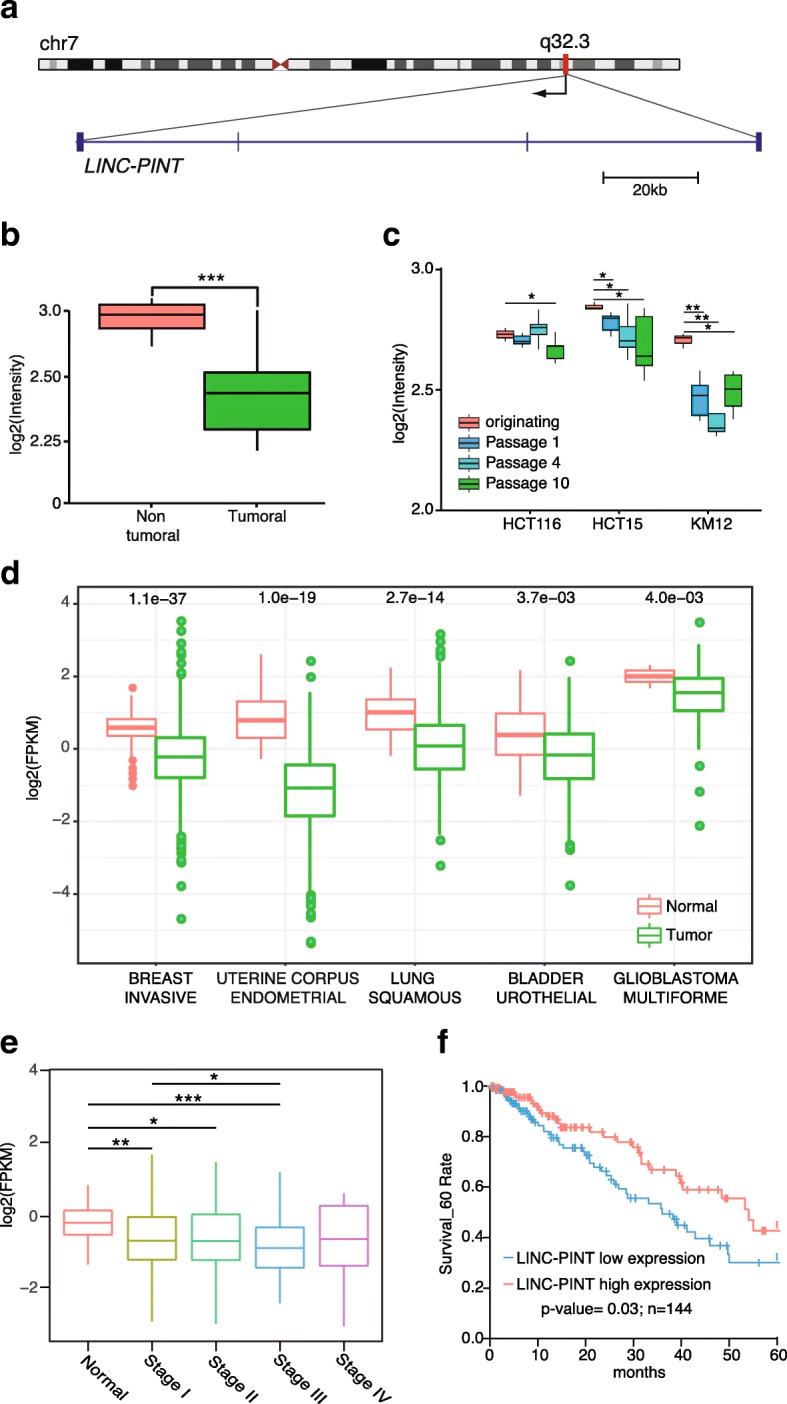
LINC-PINT is downregulated in cancer and it correlates negatively with malignancy. **a** *Schematic representation* of LINC-PINT (MKLN1-AS1 or LOC378805, FLJ43663 transcript variant 1). **b** LINC-PINT expression in colorectal (CRC) (*n* = 30) and normal tissue samples (*n* = 4). Data are obtained from GSE35602. **c** LINC-PINT expression in a collection of xenograft models at in vivo passages 1, 4, and 10 (P1, P4, and P10) along with originating cell lines (P0) GSE48433. **d** LINC-PINT expression across cancer types in non-tumoral and tumoral tissues analyzed by RNA-seq from TCGA. *P* values were calculated using Wilcoxon signed rank test. **e** LINC-PINT levels in lung adenocarcinoma tumor samples of different stages (TCGA). **f** Kaplan–Meier analyses of the correlations between LINC-PINT expression level and overall survival of 144 patients with lung adenocarcinoma (TCGA). Data are shown as mean ± SD

In a previous study, we identified and characterized Lincpint as a murine lncRNA induced by p53 that regulates cell proliferation [23]. By inspecting the syntenic region of the human genome, we identified the human ortholog of Lincpint (FLJ43663, LINC-PINT) (Fig. 1a) and showed that it is also transcriptionally regulated by p53 [23]. Indeed, it has been shown that the expression of LINC-PINT is reduced in tumors with mutations in *TP53* [24]. We also observed that the expression of LINC-PINT is decreased in tumor tissue when compared to normal tissue in independent cohorts of patients of colorectal cancer [23] (Fig. 1b and Additional file 1: Figure S1A). Moreover, the expression of LINC-PINT in colorectal cancer cell lines is further decreased when cells undergo several passages as tumor xenografts and acquire an aggressive phenotype [25] (Fig. 1c). In order to understand whether altered expression of LINC-PINT could be observed in other types of tumors, we quantified LINC-PINT expression in hundreds of normal and tumor samples from publicly available RNA-sequencing (RNA-seq) data (The Cancer Genome Atlas [TCGA], https://cancergenome.nih.gov/). This analysis showed that LINC-PINT is significantly decreased in several cancer types including breast, uterine corpus endometrial, and lung squamous cell carcinomas among others (Fig. 1d). In addition, the levels of LINC-PINT are lower in lung adenocarcinoma tumors of more advanced stage (Fig. 1e) and lower levels of the RNA are significantly associated with a diminished survival of patients (Fig. 1f), indicating an inverse correlation between the expression of LINC-PINT and the aggressiveness of the tumors.

Collectively, these observations show that the expression of LINC-PINT is inversely correlated with degree of malignancy and suggest that it could act as a tumor suppressor in different types of cancer.

### LINC-PINT inhibits the migration and invasion of cancer cells in vitro and in vivo

**Fig. 2:**
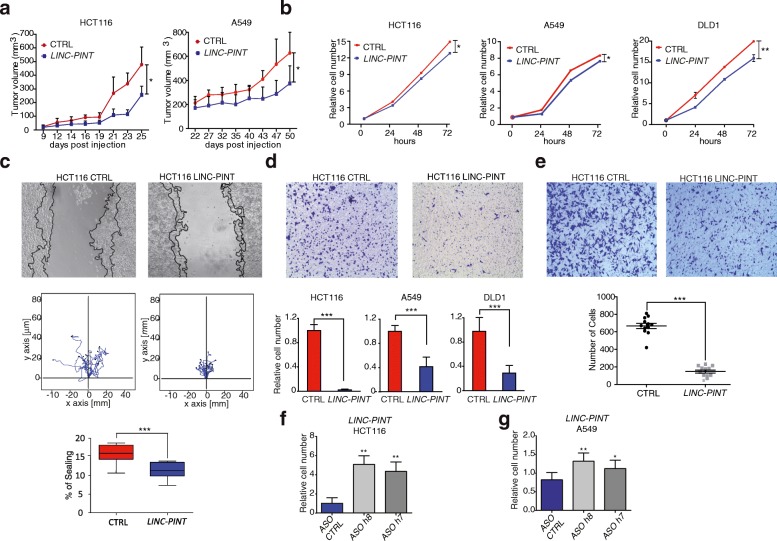
LINC-PINT overexpression inhibits the migration and invasion capability of lung and colon cancer cells. **a** Xenograft growth in nude mice injected with HCT116 CTRL cells (*n* = 6) or LINC-PINT overexpressing HCT116 cells (n = 6). Mean and standard deviation is shown. *P value < 0.05 determined by Student’s *t-*test. **b** Relative numbers at indicated times of control cells (transduced with empty vector) or cells expressing LINC-PINT. **c** Wound healing assay of control and LINC-PINT overexpressing HCT116 cells. Representative image of the invaded area as captured 12 h after the scratch (*black lines* depict the invasive front at 0 and 12 h, respectively) (*upper panel*). *Medium plots* represent single-cell tracks taken every 5 min for 12 h to ten different cells. **d** Invasion capacity of 10^5^ HCT116, A549, DLD1 CTRL cells and their equivalent LINC-PINT overexpressing cells analyzed using transwell chambers coated with Matrigel at 36 h. The number of invading cells is counted from images of five random fields per transwell. Data are shown as mean ± SD of the fold change of invading cells relative to control cell line of three independent biological replicates. **e** Cell transmigration across collagen-coated membranes. Control HCT116 and LINC-PINT overexpressing cells were allowed to migrate across collagen-coated wells for 24 h. Total number of cells in the lower side of the membrane was counted on images taken from five random fields per transwell. Data are represented as mean ± SD of migrating cells from three independent biological replicates. **f**, **g** HCT116 and A549 LINC-PINT cells were transfected with two independent antisense oligos (ASO) to knockdown LINC-PINT (ASO h5 and ASO h7), or a control ASO, and their invasion capacity was quantified as in (**d**)

To test whether the low expression of LINC-PINT favors the transformed phenotype, we investigated the effect of its gain or loss of function. First, we stably expressed the lncRNA in colorectal (HCT116) and lung (A549) adenocarcinoma cell lines, which otherwise express low levels of the lncRNA (Additional file 1: Figure S2A). To control for the cellular localization of the overexpressed LINC-PINT, we performed RNA-FISH, which showed the lncRNA localized into the nucleus with similar pattern to that of the endogenous lncRNA (Additional file 1: Figure S2B–D). When LINC-PINT overexpressing HCT116 and A549 cells were injected subcutaneously into two different types of immunocompromised mice (nude and BALB/c-Rag2/‐IL2cc), they presented a decreased ability to form tumors (Fig. 2a and Additional file 1: Figure S2E), indicating that LINC-PINT inhibits the aggressiveness of the tumor cells.

We further investigated the phenotype of the enforced expression of LINC-PINT in several cancer cell lines (colorectal HCT116 and DLD1 and lung adenocarcinoma A549, Additional file 1: Figure S2A). The expression of LINC-PINT in all of them produced a mild proliferation defect (Fig. 2b). However, the major observed phenotype was the strong impairment in cell migration and invasion capacity analyzed by wound healing (Fig. 2c) as well as matrigel (Fig. 2d) and collagen-coated transwell assays (Fig. 2e). Conversely, and consistently with the role of LINC-PINT as an inhibitor of cell invasion, the knockdown of LINC-PINT with two different antisense oligonucleotides (ASOs) resulted in an increase of the invasive capacity of the LINC-PINT cells (Fig. 2f).

**Fig. 3:**
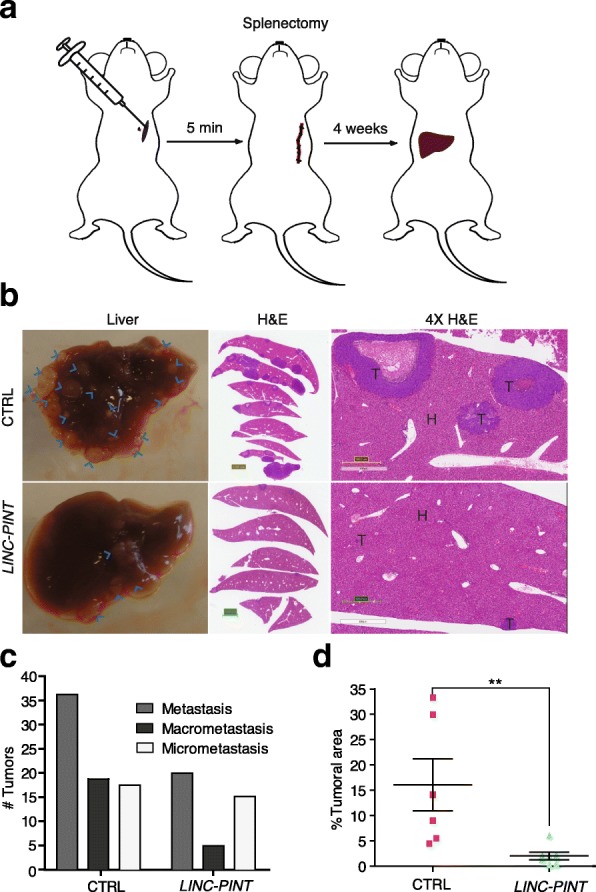
Enforced expression of LINC-PINT decreases metastasis initiation of CRC cells. **a**–**d** Intrasplenic mice inoculation with 2.5 × 10^5^ HCT116 control cells (CTRL) (*n* = 6) or LINC-PINT overexpressing HCT116 cells (LINC-PINT) (*n* = 6). **a** *Schematic representation* of liver metastasis mice model induced by intrasplenic injection of colon cancer cells. **b** Representative *pictures* of liver metastases (*blue arrows*) at time of sacrifice (*left*), hematoxylin and eosin (H&E)-stained sections scanned on an Aperio Scan Scope AT (*middle*) and 4X magnifications of H&E slides; healthy and tumoral tissue is pointed out with (H) and (T), respectively. **c** Quantification of number of liver metastasis, micrometastases ≤ 2 mm and macrometastases ≥ 2 mm. **d** *Graphic representation* of percentage of tumoral area per mice liver quantified on Aperio Image Scope (Leica Biosystems, Buffalo Grove, IL, USA) (*P < 0.05, *P* < 0.01 two-tailed Student’s *t*-test)

We next tested whether LINC-PINT was also able to inhibit cell invasiveness in vivo, using a mouse model of liver metastasis [26]. For this, we inoculated HCT116 cells overexpressing LINC-PINT or control cells into the portal circulation of mice through intrasplenic injection followed by splenectomy after 5 min and quantified the liver metastases in the mice four weeks post injection (Fig. 3a). While both control and LINC-PINT cells were able to metastasize to the liver, the number of macro- and micro-metastases was significantly decreased in LINC-PINT overexpressing cells (Fig. 3b–d). These results show that LINC-PINT not only inhibits the ability of cells to invade in vitro, but also reduces the engraftment potential of the cells in vivo.

### A conserved RNA sequence element of LINC-PINT is required for inhibiting cell invasion

**Fig. 4:**
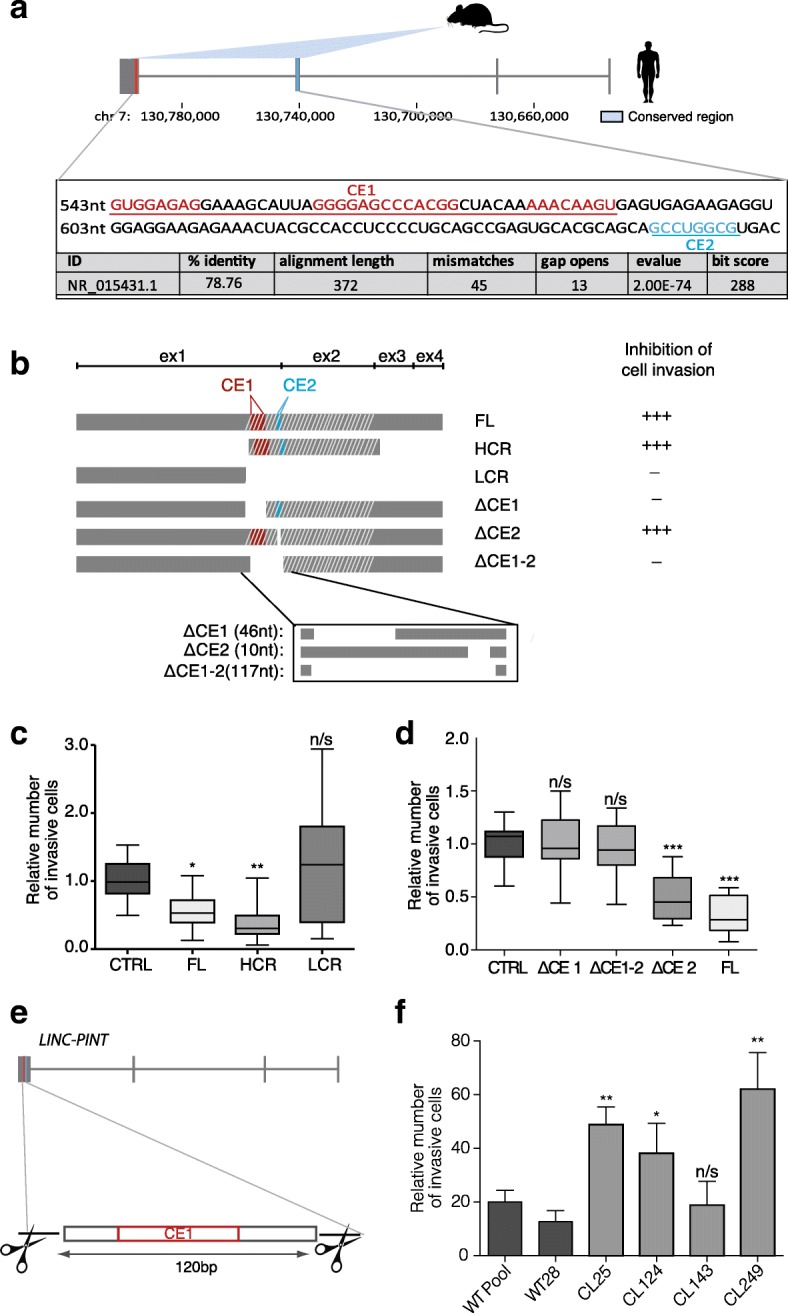
A highly conserved short region of LINC-PINT is required for its function. **a** *Schematic representation* of alignment signatures found for mouse *Lincpint* and the orthologus human LINC-PINT using *slncky* Evolution Browser [10]; the conserved region between mouse and human is emphasized and the conserved sequences between mammals [12] are underlined in *red* (CE1) and *blue* (CE2). Conservation values of BLAST are summarized in the bottom table. **b** *Schematic representation* of FL LINC-PINT clone and the LINC-PINT mutants; HCR, LCR, CE1 deletion (ΔCE1), CE2 deletion (ΔCE2), and CE1-2 deletion (ΔCE1-2) (*left*), and the invasion phenotype observed upon their expression. **c**, **d** Invasion assay performed as in Fig. 2d in HCT116 cells expressing the indicated forms of LINC-PINT or an empty vector (CTRL). **e** *Schematic* of LINC-PINT fragment deletion by CRISPR-Cas9. **f** Invasion phenotype of HCT116 cellular clones with homozygous deletion of CE1 sequence (CL25, CL124, CL143, and CL249) or normal cells (WT pool and WT28). Significance was determined by Mann–Whitney U test (*P < 0.05, **P < 0.01)

lLINC-PINT is not only found in humans, but it is present in other vertebrates with sequence-similar homologs throughout mammals and positionally conserved lncRNAs in birds (Additional file 1: Figure S3A). Moreover, we showed that the transcriptional regulation of LINC-PINT by p53 is conserved between mouse and human, suggesting the functional conservation of this lncRNA [23]. Consistently with this idea, the enforced expression of murine Lincpint in human cells had similar effect as the human lncRNA, resulting in a significant decrease of cell invasion, a phenotype that could be rescued when the expression of the murine RNA was specifically inhibited with ASO transfection (Additional file 1: Figure S3B and S3C). We therefore reasoned that the activity of LINC-PINT was dependent on RNA sequences conserved between mouse and human. Indeed, a sequence comparative analysis between the murine and human transcripts showed high homology in the region between nucleotides 535 and 924 of human LINC-PINT (e-value 2.00E-74) (Fig. 4a). To test the functionality of this region of the lncRNA, we first generated a truncated form of LINC-PINT that lacks the conserved nucleotides and only contains the 516 nt 5′ of the lncRNA (lowly conserved region [LCR], Fig. 4b). When evaluated in the invasion assay, in contrast to the full-length (FL) LINC-PINT, the LCR had no effect in the invasion capacity or proliferation of HCT116 cells (Fig. 4b, c), although it was expressed at similar levels (Additional file 1: Figure S3D). We next investigated whether the region conserved between mouse and human was sufficient to mediate the function of the lncRNA in cell invasiveness. We therefore generated a mutant that only contains this region of LINC-PINT (highly conserved region [HCR], 389 nts, Fig. 4b). When stably expressed in cells, this conserved fragment of LINC-PINT (HCR) could reduce the invasiveness to a level even lower than that of the FL LINC-PINT (Fig. 4b, c, Additional file 1: Figure S3D), indicating that this fragment of LINC-PINT is sufficient for its activity in the context of invasion.

It has been proposed that the function of some lncRNAs is dependent on short sequence elements conserved across species [27]. We hypothesized that such elements may be contained in the functional fragment that we identified in LINC-PINT. To be able to pinpoint the relevant sequences, we expanded the LINC-PINT cross-species analysis to 17 species of mammals (opossum included). The comparative analysis identified several short conserved elements inside the functional HCR of LINC-PINT (Fig. 4a). We then performed different deletions of these sequences generating ΔCE1 mutant, which lacks CE1, a 46-nt fragment with three short conserved motifs (Fig. 4a and b); ΔCE2, which lacks a more distant 8-nt motif; and ΔCE1-2, a 117-nt deletion that eliminates all of them (Fig. 4b). We then tested the effect of these mutants in HCT116 cells. The experiments revealed that the lack of CE1 (deletions ΔCE1 and ΔCE1-2) totally abolished the effect of LINC-PINT in invasiveness (Fig. 4d, Additional file 1: Figure S3E) and tumor formation (Additional file 1: Figure S3F). In contrast, the deletion of CE2 did not affect the ability of LINC-PINT to reduce cell invasiveness (Fig. 4d, Additional file 1: Figure S3E). These results suggest that the role of LINC-PINT is highly dependent on CE1 sequence.

To further confirm this observation and to avoid the ectopic expression of the lncRNA mutants, we used CRISPR-Cas9 genome editing to generate a homozygotic deletion of a 120-nt fragment of endogenous LINC-PINT (containing CE1 but not CE2) in HCT116 cells (Fig. 4e and Additional file 1: Figure S3G). Consistently with our previous findings, three out of four CE1-deficient clones showed increased invasiveness when compared to LINC-PINT wild type (WT) cells (as individual or pooled WT clones) (Fig. 4e). Moreover, the deletion of this sequence increased the capacity of the cells to form tumors in vivo (Additional file 1: Figure S3H). Altogether, these data demonstrate that the CE1 sequence is required for the inhibition of cell invasion mediated by LINC-PINT.

### LINC-PINT suppresses the expression of an invasion signature

**Fig. 5:**
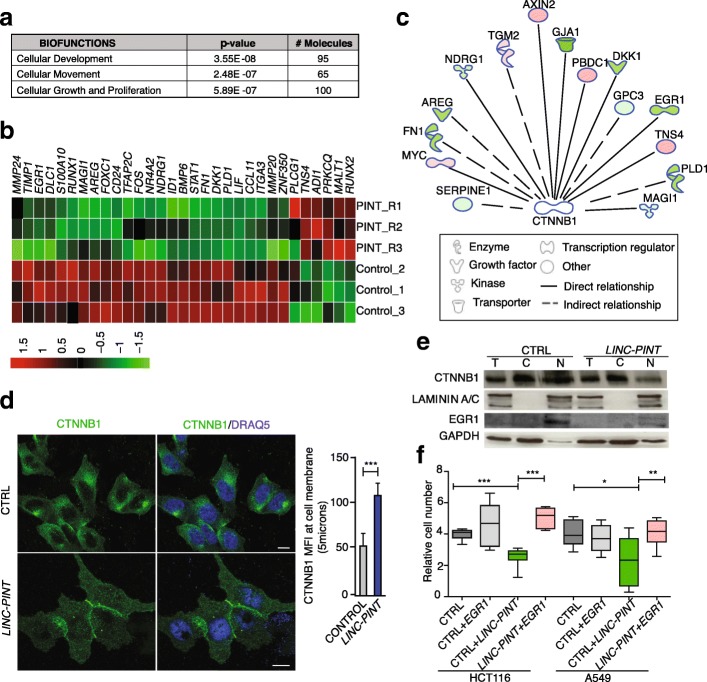
LINC-PINT represses the expression of an invasion signature and induces CTNNB1 translocation. **a** Biological functions associated with genes differentially expressed upon LINC-PINT overexpression in HCT116 cells. **b** *Heatmap* representation of genes differentially expressed (DE) in HCT116 overexpressing LINC-PINT vs. HCT116 CTRL cells, involved in tumor cell adhesion, as defined by IPA (*green*, downregulation; *red*, upregulation). **c** Connection between CTNNB1 and genes regulated by LINC-PINT involved in cell movement and proliferation as predicted by IPA. **d** Immunoflorescence images of CTNNB1 (*green*) and DRAQ5 (*blue*, nuclear specific marker) in control cells (CTRL) and LINC-PINT overexpressing HCT116 cells (LINC-PINT). Scale bars: 20 μm (*left*). The fluorescence intensities of CTNNB1 are quantified by tracing a scanning line of 5 μm across the plasma membrane of the cell (*right*). **e** Subcellular fractionation and western blot analysis performed in HCT116. Three different fractions are loaded; total cell fraction (T), cytoplasmic fraction (C), and nuclear fraction (N) and probed for CTNNB1 and EGR1. GAPDH was used as cytoplasmic marker and LAMININ A/C as nuclear marker. **f** EGR1 overexpression restores invasive capacity of LINC-PINT overexpressing A549 and HCT116. Cells were either transduced with an empty vector (CTRL) or with LIC-PINT (LINC-PINT) and then transiently transfected to overexpress EGR1 (CTRL + EGR1 or LINC-PINT + EGR1). Data are from three biological replicates represented as mean ± SD of the fold change of invading cells. Significance was determined by one tail *t*-test (*P < 0.05, **P < 0.01, ***P < 0.001)

The gain of function of LINC-PINT has a strong impact on the invasive capacity of cancer cells. In order to determine the cellular pathways involved, we extracted total RNA from HCT116 overexpressing LINC-PINT and control cells and performed gene expression analyses by microarray. We found 533 genes differentially expressed (*P* value < 0.01), of which 233 were upregulated and 301 downregulated in LINC-PINT overexpressing cells compared to control cells (Additional file 2: Table S1). The gene set was found enriched in different biological functions, but among the most significant were cellular development, cellular movement, and cellular growth and proliferation (Fig. 5a and Additional file 3: Table S2). When the tumor cell adhesion network was analyzed in detail, we found several genes downregulated upon LINC-PINT overexpression related with cancer cell migration capacity, such as Early Growth Response 1 (EGR1), Phospholipase D1 (PLD1), Leukemia inhibitory factor (LIF), FBJ osteosarcoma oncogene (FOS), SERPINE1, Fibronectin1 (FN1), or Integrin alpha 3 (ITGA3) (Fig. 5b and Additional file 1: Figure S4A). These gene expression changes, which are consistent with the decreased proliferation and invasion capacity of the cells, were independently validated by qRT-PCR (Additional file 1: Figure S4B). Interestingly, the analysis performed by Ingenuity Pathway Analysis (IPA) (QIAGEN Inc., https://www.qiagenbioinformatics.com/products/ingenuity-pathway-analysis) [28] indicated that several of the genes regulated by LINC-PINT are functionally connected with beta-catenin (CTNNB1), a key factor in cell growth and adhesion [29] (Fig. 5c). Since the messenger RNA (mRNA) levels of beta-catenin were not altered and the protein function relies on its subcellular localization [30], we performed immunofluorescence to investigate beta-catenin localization in our cellular conditions. We observed that enforced expression of LINC-PINT induced a translocation of beta-catenin to the cytoplasmic membrane of the cells (Fig. 5d and Additional file 1: Figure S4C). Consistently, subcellular fractionation followed by western blot analysis showed lower levels of beta-catenin in the nuclear fraction of LINC-PINT overexpressing cells, concomitant with the reduction of mRNA and protein levels of the beta-catenin regulator EGR1 [31, 32] (Fig. 5e). In agreement with these observations, several of EGR1 direct target genes (ENDNOTE, Additional file 1: Figure S5A) appear downregulated (Additional file 1: Figure S5B), and the association of EGR1 to their promoters is decreased in LINC-PINT-overexpressing cells (Additional file 1: Figure S5C). Moreover, we found that the overexpression of EGR1 is able to rescue the loss of invasive phenotype caused by the enforced LINC-PINT expression (Fig. 5f). This indicates that the inhibition of EGR1 mediates, at least in part, the less invasive phenotype caused by LINC-PINT in colorectal and lung adenocarcinoma cells. Collectively our data show that LINC-PINT regulates the expression of genes that contribute to the ability of cancer cells to migrate, inducing the subcellular translocation of beta-catenin.

### PRC2 mediates the LINC-PINT-dependent silencing of invasion genes

**Fig. 6:**
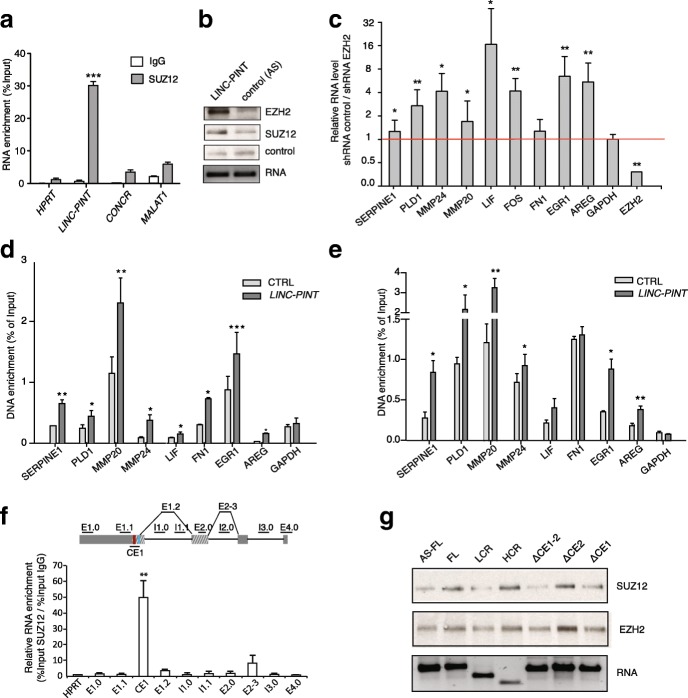
PRC2 mediates LINC-PINT-dependent silencing of invasion genes. **a** Level of enrichment in SUZ12 immunoprecipitates of the indicated coding and non-coding RNAs in HCT116 cells. IgG is used as control. **b** EZH2 and SUZ12 proteins bound to LINC-PINT or antisense RNA (control RNA) when incubated with nuclear extracts. An unspecific cross-reacting protein is shown as control. **c** Expression changes of genes in LINC-PINT overexpressing HCT116 cells upon EZH2 depletion by shRNA. **d**, **e** SUZ12 (d) or H3K27me3 (e) enrichment in promoter regions of LINC-PINT-regulated genes in control or LINC-PINT HCT116 cells. Enrichment values are relative to the input. Mean ± SD of three qPCR replicates of a representative experiment are shown. **f** FA crosslinking and immunoprecipitation (fRIP) of SUZ12-bound LINC-PINT in HCT116. qRT-PCR identifies the LINC-PINT region bound by PRC2 in vivo. The *scheme* represents the location of the oligos along LINC-PINT transcript; *E* exon, *I* intron. **g** RNAs corresponding to FL or different fragments of LINC-PINT or its antisense sequence (AS-FL) were obtained by in vitro transcription. Their interaction with recombinant purified PRC2 was tested by RNA pull-down and SUZ12 and EZH2 was detected by western blot

We set to investigate how LINC-PINT causes the downregulation of the pro-invasion gene signature. Interestingly, several of the genes of this signature are also downregulated when mouse Lincpint is expressed in human cells (Additional file 1: Figure S6A), suggesting that their inhibition is caused by a mechanism shared by the murine and the human form of the lncRNA. We had previously shown that the murine ortholog of LINC-PINT (lincPint) interacts with the PRC2 and it is required for the efficient targeting and repression of a subset of genes by this protein complex [23]. In addition, an independent study had identified the human LINC-PINT as a nuclear lncRNA that interacts with PRC2 in human fibroblasts [15]. We then confirmed that LINC-PINT and PRC2 interact in human cells of different origins, including normal and cancer cell lines, by detecting specific enrichment of LINC-PINT in PRC2 immunoprecipitates (Fig. 6a and Additional file 1: Figure S6B–D), as well as the reciprocal RNA pulldown experiments (Fig. 6b). Furthermore, LINC-PINT and PRC2 are likely direct interactors, since their endogenous association was detected using either ultraviolet (UV) or formaldehyde crosslinking followed by stringent washes (Additional file 1: Figure S6C and D), as well as when using purified PRC2 and LINC-PINT incubated in vitro (Additional file 1: Figure S6E). We therefore hypothesized that the activity of LINC-PINT may be, at least in part, related to PRC2. Indeed, several of the genes that compose the invasion signature inhibited by LINC-PINT (Fig. 5b) are marked with H3K27me3 in different cell types (Additional file 1: Figure S6F), indicating that they are potentially regulated by PRC2. We then investigated if the observed expression changes induced by LINC-PINT on these genes were mediated by PRC2. To test this, we inhibited the expression of PRC2 in LINC-PINT-overexpressing HCT116 cells by using an shRNA against EZH2, the catalytic subunit of the complex, and analyzed the expression of several of the genes by reverse transcription quantitative polymerase chain reaction (qRT-PCR). The expression levels of the majority of the genes analyzed (7/8) present in the invasion signature, were induced by PRC2 knockdown, suggesting that their silencing by LINC-PINT is PRC2-dependent (Fig. 6c). To further explore if PRC2 associates to these genes in a LINC-PINT-dependent manner, we performed chromatin immunoprecipitation (ChIP) in normal HCT116 and LINC-PINT HCT116 cells using an antibody for the PRC2 core subunit SUZ12. The ChIP-qPCR showed that the binding of SUZ12 to all of the promoters was significantly increased when LINC-PINT had elevated expression (Fig. 6d). Concomitant with the increased PRC2 occupancy, almost all the gene promoters analyzed (6/8) showed a significant increase in the levels of H3K27me3, the epigenetic modification catalyzed by PRC2 (Fig. 6e). In conclusion, these results suggest that LINC-PINT acts together with PRC2 to silence the expression of genes involved in cell invasion.

Next, to investigate what region of LINC-PINT is involved in the interaction with PRC2, we applied the RIP protocol after crosslinking with formaldehyde (fRIP) combined with RNA fractionation. Following fractionation and SUZ12 immunoprecipitation, the RNA fragments of LINC-PINT bound by PRC2 were detected by qRT-PCR with primers tiled along LINC-PINT sequence (Fig. 6f). Interestingly, we observed the highest enrichment with primers mapping at the CE1 region (Fig. 6f), suggesting that this portion of LINC-PINT mediates its interaction with PRC2. In agreement with our findings, the analysis of the CE1 sequence revealed several motifs that can potentially form G-quadruplex structures (Additional file 1: Figure S6F), recently shown to be preferentially bound by PRC2 [22]. Moreover, two of the three short-conserved sequence motifs contained within CE1 (Fig. 4a) were also found highly enriched in EZH2 (*P* values = 1.35 × 10^–34^ and 2.9 × 10^–26^) and SUZ12 (*P* values = 3.41 × 10^–44^ and 3.6 × 10^–34^) fRIP-seq experiments preformed in K562 cells [33].

Finally, we further tested whether the ability of LINC-PINT to bind PRC2 in vitro was dependent on CE1 sequence. For this, we synthesized the different mutant forms of LINC-PINT (Fig. 4b), as well as the FL LINC-PINT and the antisense full length (AS-FL) as control. We then incubated equimolar amounts of each of the RNAs with purified PRC2 complex and performed RNA pull-downs. The results confirmed that the full length LINC-PINT and the HCR mutant bind PRC2 with the highest affinity despite HCR being the shortest of the RNA mutants tested (Fig. 6g). Moreover, while the deletion of CE2 had no effect, the deletion of CE1 resulted in decreased binding (Fig. 6g). These observations, together with the functional analysis of the mutant forms of LINC-PINT (Fig. 4), strongly support the notion that the interdependence between LINC-PINT and PRC2 relies on the conserved CE1 sequence. Collectively, these results show that LINC-PINT, which is downregulated in several types of cancer, included colorectal and lung cancer, contributes to the PRC2-dependent silencing of an invasion gene signature mediated by a sequence element conserved in mammals.

## Discussion

The capacity of migration of cancer cells is essential for the process of metastasis, in which the tumor spreads from the place where it first arose to distant locations in the body. Therefore, investigating the molecular mechanisms that regulate metastasis may provide helpful insights into the development of efficient diagnosis and therapeutic strategies. Here we show how LINC-PINT, which is kept at low levels in tumors, acts as an inhibitor of this major cancer hallmark. Notably, p53 is known to constrain the metastasic capacity of cancer cells [34] and LINC-PINT is transcriptionally regulated by p53 [23]. Therefore LINC-PINT represents an additional effector of the broad tumor suppressor activities of p53. Although several other lncRNAs have been reported to promote cancer metastasis, such as the well-characterized MALAT1 [35] and HOTAIR [36], less evidence exists of lncRNAs acting as inhibitors of this process [37]. For instance, low expression of PTENP1 was related with decreased cell invasion and poor prognosis in several cancer types including melanoma [38] and head and neck squamous cell carcinomas [39]. While PTENP1 acts post-transcriptionally competing with PTEN for the binding of inhibitory microRNAs [40], LINC-PINT is remarkably enriched in the nucleus of the cells. This is consistent with its involvement in the transcriptional modulation of several upstream regulators of the invasive phenotype of cancer cells.

We have shown that the murine lincpint presents several similarities with its human ortholog, such as the regulation by p53 [23] and the ability to interact with PRC2. We thus used a cross-species conservation analysis combined with functional validations to be able to understand the mechanism of LINC-PINT function. Our data show that a truncated version of LINC-PINT (HCR) contains all the elements necessary to inhibit the migration of cancer cells. Moreover, we found a short sequence motif (CE1) that is highly conserved in mammals and required for LINC-PINT function. Interestingly, this motif is also required for the interaction of LINC-PINT with PRC2. Beyond our own experimental data, the preferential binding of PRC2 for CE1 sequence is supported by independent lines of evidence; for instance, the sequence motifs contained within CE1 are strongly enriched in fRIP-seq PRC2 data [33] and its sequence characteristics are consistent with recent findings that determine the affinity of PRC2 for G-rich and G-quadruplex forming RNA regions [22].

A model where individual lncRNAs act as guides for PRC2, conferring it with specificity for gene targets was proposed few years ago based on HOTAIR [41] and Xist [14] seminal works. Arguments against this model have been raised by studies showing the low specificity of the binding of PRC2 to RNA [21, 22], the mutually exclusive binding between PRC2 and RNA or chromatin [42, 43] or the inhibitory effect of RNA on PRC2 [42, 43]. Our study indicates that the function of LINC-PINT is dependent on a highly conserved sequence that specifically binds to PRC2 and that PRC2 is required for the silencing of gene targets leading to inhibition of cell invasion. Interestingly, LINC-PINT is not associated to the chromatin, but it is mainly present in the soluble fraction of the nucleus (Additional file 1: Figure S2D), which suggests that LINC-PINT interacts with chromatin-free PRC2. Furthermore, the genes co-repressed by LINC-PINT and PRC2 are transcriptional targets of EGR1 and the binding of EGR1 to their promotes decreases in conditions of LINC-PINT overexpression (Additional file 1: Figure S5A–C). This is in agreement with a previous report showing that the downregulation of EGR1 gene targets is accompanied with epigenetic silencing by PRC2, which prevents EGR1 re-association [44]. Taking into account all these data, we propose a model where LINC-PINT could act as a DNA decoy providing PRC2 to the proximity of active promoters that are bound by the transcriptional activator EGR1. The transcriptional activator is released from the promoter, while PRC2 would be released from LINC-PINT to bind to the promoter for silencing. The silencing by PRC2 may be sufficient to avoid EGR1 re-association to the chromatin, although it is also possible that LINC-PINT itself acts as a specific EGR1 inhibitor. Future work will help to further delineate LINC-PINT mechanism and possibly other PRC2-interacting lncRNAs.

## Conclusions

Our findings demonstrate the involvement of the downregulation of LINC-PINT in cancer progression and tumor malignancy. Moreover, they support a conserved functional co-dependence between LINC-PINT and PRC2 that counteracts gene activation by EGR1. It leads us to propose a new mechanism where the lncRNA regulates the availability of soluble PRC2 at the proximity of specific genomic regions, suggesting that the interplay between lncRNA and DNA binding proteins may be as relevant as protein–protein interactions in the regulation of gene expression.

## Methods

### RNA preparation and RT-qPCR

Total RNAs were extracted from tumors and adjacent normal tissues or cultured cells using Trizol reagent (Invitrogen) following the manufacturer’s protocol. RT and qPCR kits (Invitrogen) were used to evaluate the expression of LINC-PINT in tissue samples and cultured cells. RT-PCR was performed in quatriplicate and the relative expression of *LINC-PINT* was calculated using the comparative cycle threshold (CT) (2 − ΔΔCT) method with glyceraldehyde-3-phosphate dehydrogenase (GAPDH) or Hypocanthine Phosphoribosyltransferase (HPRT) as the endogenous control to normalize the data.

### Vector construction and retrovirus infection

The cDNA of *LINC-PINT* sequence (BC130416) was subcloned into the pBABE-puro vector for retrovirus production. Then HCT116, A549, and DLD1 cells were infected and selected with 1.5 μg/mL of puromycin for 72 h.

### Antisense oligo (ASO) transfection

To generate *LINC-PINT* knockdown HCT116 cells, two independent ASOs that target *LINC-PINT* or ASO control were synthesized by Ionis Pharmaceuticals®. ASOs where added to the medium for cell free uptake at final concentration of 625nM for HCT116-PINT cells and 5 mM for A549-PINT cells. ASO sequences are shown below.

### Tumor analysis

Gene expression was determined from RNA-seq data available through the TCGA database (https://cancergenome.nih.gov/). The aligned reads were assigned and quantified using Cufflinks v2.2.1. *LINC-PINT* expression was compared in each cancer type between normal tissue samples and primary tumor samples. Statistical significance was determined by unpaired Student’s *t*-test.

RNA from tumors of patients with colorectal and lung adenocarcinoma were obtained from the Basque Biobank for Research-OEHUN and the Navarra University Hospital.

### Microarray analysis

For gene expression profiling, total RNA was extracted and hybridized to Affymetrix Human Transcriptome Array 2.0. Background correction and normalization were done using RMA (Robust Multichip Average) algorithm [45] using Affymetrix Power Tools. After quality assessment, a filtering process was performed to eliminate low-expression probe sets. Applying the criterion of an expression value > 16 in two samples for each experimental condition, 41,697 probe sets were selected for statistical analysis. R and Bioconductor were used for preprocessing and statistical analysis. LIMMA (Linear Models for Microarray Data) [46] was used to find out the probe sets that showed significant differential expression between experimental conditions. Genes were selected as significant using a *P* value > 0.01. The biological knowledge extraction was complemented through the use of Ingenuity Pathway Analysis (QIAGEN Inc., https://www.qiagenbioinformatics.com/products/ingenuity-pathway-analysis).

### RIP-Seq and sequence enrichment analysis

Formaldehyde RNA immunoprecipitation (fRIP-Seq) raw sequencing data of PRC2 complex proteins Ezh2 and Suz12 were downloaded from GEO database (GSE67963) [33]. Sequencing reads were aligned to the human genome assembly hg19 using Bowtie v2.1.0 [47] and genes were quantified using FeatureCounts v1.5.0 [48]. In each dataset, the gene enrichment was calculated using R/Bioconductor package *limma* using voom [49] normalization. The occurrences of each of PINT functionally relevant sequences were determined among the transcripts of the enriched genes (B > 0, logFC > 0), and its significance was calculated by means of a hypergeometric test compared to the human transcriptome.

### Cell proliferation assays

For proliferation analysis, 2000 cells were plated per well in 96-well plates and the CellTiter96 Aqueous Non-Radioactive Cell Proliferation Assay (MTS) kit (Promega®) was used. Cell viability was assessed every 24 h following the manufacturer’s protocol. All experiments were performed in triplicate.

### Nuclear fractionation

Subcellular fractionation, a total of 10^7^ cells were trypsinized and washed once with cold PBS, aliquoted into two tubes, and collected by centrifugation at 1000 *g* for 5 min at 4 °C. One cell pellet represented the whole-cell extract, while the other one was processed for the remaining subcellular fractions. Both pellets were resuspended in 500 μL of Buffer A (10 mM Tris-HCl, pH 7.5, 1.5 mM MgCl_2_, 140 mM NaCl, 0.05% IGEPAL supplemented with protease inhibitor cocktail and SuperaseIN 10 U ml^−1^), incubated for 10 min on ice, and kept for subsequent RNA extraction. A total of 500 μL of Buffer A plus sucrose (10 mM Tris-HCl, pH 7.5, 1.5 mM MgCl2, 140 mM NaCl 0.5% IGEPAL, 50% Sucrose) was added to the bottom of a clean Eppendorf tube and the upper phase (whole-cell extract resuspended in Buffer A) was gently added to this tube preventing the mix of the two phases and centrifuged for 10 min at 4 °C and 12,000 *g* to obtain nuclear and cytoplasmic fractions. Around 500 μL of the upper phase (cytoplasmic fraction) was collected and the rest was discarded, leaving the pellet (nuclear fraction). Total nuclear fraction was resuspended in 500 μL of Buffer B (10 mM Tris, 100 mM NaCl, 1 mM EGTA, 300 mM sucrose, 0.5 mM NaVO_3_, 50 mM NaF, 1 mM phenylmethylsulphonyl fluoride, 0.5% triton X-100, protease inhibitor cocktail, and SuperasIN) and incubated for 10 min on ice to permeabilize the cells. To separate nuclear soluble from nuclear insoluble fraction, the sample was centrifuged at 2000 *g* for 5 min at 4 °C and the supernantant (nuclear *s*oluble fraction) and the pellet (nuclear insoluble/chromatin fraction) was collected. The nuclear insoluble fraction was resuspended in Buffer A and finally 1 mL of Trizol was added to all tubes for subsequent RNA extraction.

### RNA FISH

RNA FISH for *LINC-PINT* detection was performed using a pool of 48 fluorescent probes purchased from Stellaris Biosearch Technologies by following manufacturer’s protocol.

### Crosslinking immunoprecipitation (CLIP)

The CLIP protocol was performed as previously described [50] with the following modifications: LINC-PINT overexpressing HCT116 cells were UV cross-linked (254 nm) with 4000 mJ/cm^2^. Lysates were prepared as previously indicated and sonication was used to fragment the RNA to 200–400 nt. RNA immunoprecipitation was performed for endogenous SUZ12 (Abcam cat# 12073) in LINC-PINT HCT116 cells.

### Formaldehyde-crosslinked RNA immunoprecipitation (fRIP)

10^7^ cells were crosslinked with 0.5% formaldehyde, and incubated with 0.125 M of glycine for 5 min to quench the formaldehyde and terminate the cross-linking reaction. Cells were resuspended in 2 mL PBS, 2 mL nuclear isolation buffer (1.28 M sucrose; 40 mM Tris-HCl pH 7.5; 20 mM MgCl2; 4% Triton X-100), and 6 mL water on ice for 20 min (with frequent mixing). Nuclei were pelleted by centrifugation at 2500 G for 15 min. The nuclear pellet was resuspended in 1 mL RIP buffer (150 mM KCl, 25 mM Tris pH 7.4, 5 mM EDTA, 0.5 mM DTT, 0.5% NP40, 9 ug/mL leupeptin, 9 ug/mL pepstatin, 10 ug/mL chymostatin, 3 ug/mL aprotinin, 1 mM PMSF, 100 U/mL SUPERASin; Ambion). Resuspended nuclei were split into two fractions of 500 μL each (for Mock and IP) and were mechanically sheared using a dounce homogenizer with 15–20 strokes. Nuclear membrane and debris were pelleted by centrifugation at 13,000 RPM for 10 min. Antibody to Suz12 (Abcam cat# 12073) and IgG as a negative control were incubated overnight at 4 °C with gentle rotation. A total of 50 μL of protein A/G magnetic beads were added and incubated for 2 h at 4 °C with gentle rotation. Beads were collected using a magnet, removing the supernatant, and beads were resuspended in 500 μL RIP buffer and repeated for a total of three RIP washes, followed by one wash in PBS. Beads were incubated for 45 min at 70 °C to reverse crosslinking. Beads were then resuspended in 0.5 mL of Trizol.

### RNA pull-down

RNA pull-down was performed as previously described [51]. Biotinylated RNA was incubated with nuclear extracts or recombinant PRC2 (Diagenode® cat# 31387) and streptavidin magnetic beads were used.

### Chromatin immunoprecipitation (ChIP)-qPCR

Cells were crosslinked with 1% of formaldehyde diluted in PBS for 10 min at room temperature; cells were then incubated with 0.125 M of glycine for 5 min to quench the formaldehyde and terminate the cross-linking reaction. Cells were incubated with cell lysis buffer (5 mM Tris pH 8.0, 85mMKCl, 0.5% NP-40, supplemented with Roche protease inhibitor cocktail). Nuclear pellet was collected by centrifugation and resuspended in RIPA buffer (1 × PBS, 1% NP-40, 0.5% Na-deoxycholate, 0.1% SDS supplemented with Roche protease inhibitor cocktail), then the chromatin is sheared using a Diagenode bioruptor instrument with the following conditions: eight cycles 30″ON/30″OFF, 4 °C, which typically results in shear sizes for DNA between 0.5 kb and 0.2 kb. Sheared chromatin was incubated overnight with 3–6 ug of H3K27me3 ab (Abcam #6002), SUZ12 ab (Abcam#12073), EGR1 ab (Santa Cruz#110), or negative control IgG (Cell Signalling #2729). Then, chromatin was incubated with Dynabeads® (Invitrogen) for 2 h beads. After that beads were washed five times with LiCl wash buffer (100 mM Tris pH 7.5, 500 mM LiCl, 1% NP-40, 1% Na-deoxycholate) and 1x with TE (10 mM Tris pH 7.5, 0.1 mM Na_2_EDTA). The ChIPed DNA was eluted for 1 h at 65 °C in Elution buffer (1% SDS, 0.1 M NaHCO_3_), reverse X-linked, purified, and analyzed by qPCR.

### Mouse xenograft

1 × 10^6^ *LINC-PINT* overexpressing HCT116 (*LINC-PINT*) and HCT116 transduced with an empty vector (CTRL) cells in an exponential growth phase were subcutaneously injected in the flanks of 6–7-week-old female BALB/c-Rag2/-IL2cc/immunodeficient mice (*n* = 6 per experimental condition) and female athymic nude mice (*n* = 6 per experimental condition). For the mouse xenograft experiment with A549 cell line, 5 × 10^6^ cells were injected in BALB/c-Rag2/-IL2cc/immunodeficient mice (*n* = 6 per experimental condition). Injection specifications, 50 uL of cells with the amount of cells required for one mouse injection is mixed with the same amount of Matrigel®; 100 μL of the resultant mix is injected in each mouse. Tumor size was measured externally using a precision caliper and tumor volume (V) was calculated using the following equation: V = π/6 × width^2^ × length. The tumor growth was measured over 25 days every two days.

### Liver metastases mice model

HCT116 CTRL and HCT116 LINC-PINT cells were grown to confluence and harvested as described above for subcutaneous injection and resuspended in PBS at a concentration of 5 × 10^6^ cells/mL. BALB/c-Rag2/-IL2cc/immunodeficient mice (*n* = 6 per experimental condition) were anesthetized with isofluorane by inhalation and the spleen through a left flank incision. 2.5 × 10^5^ cells in 50 μL were slowly injected into the spleen and as the needle is remove from the spleen, a sterile cotton swabs avoided that cells came out. After 5 min the spleen is disconnected from the body’s blood supply and it was removed by cauterization; the surgical openings were then closed using sutures. All animals were killed when the first mouse with an enlarged liver could be palpated (day 28). The liver was excised and fixed in 3.7–3.8% hydrous formaldehyde solution before H&E-stained section preparation. Each preparation was scanned on an Aperio Scan Scope AT. After that, the number of liver macrometastasis ≥ 2 mm and micrometastases on Aperio Image Scope (Leica Biosystems, Buffalo Grove, IL, USA) was quantified.

### Wound healing

3 × 10^5^ cells were plated on a 24-well culture plate (Corning Costar) in cell culture media containing 10% FBS and allowed to growth to confluence. Afterwards, cells were serum starved for 4 h and the monolayer was scratched using a pipette tip. The cell migration into wound area was monitored at 0 and 12 h after wounding, using a Leica DMIL LED inverted microscope (Leica Microsystems). The percentage of healed surface at each time point related to time cero was calculated using Fiji software. Data were normalized to the values obtained in CTRL cells at each time point. Three independent experiments were performed and 12 different fields per group were analyzed. Insets show representative bright-field images at 24 h post scratch; black lines highlight the initial (t = 0 h) and final (t = 12 h) wound edges.

Wound healing in vivo assay, cells were imaged every 5 min for 12 h and ten single-cell tracks are superimposed at the origin with the following variables: total distance of migration (μm) and percentage of sealing were quantified using Fiji software.

### Transwell migration and invasion assay

10^5^ HCT116 cells were plated onto the upper side of 8-μm pore-size transwell inserts (Corning) previously pre-coated with type I rat tail collagen. Cells were cultured in serum free media 4 h before allowing cell migration towards complete cell media at 37 °C for 14 h. Afterwards, cells were fixed in 4% formaldehyde for 15 min and the upper side of the insert was thoroughly wiped off with cotton swabs. The lower part of the insert was stained with 0.5% crystal violet. Images were captured using a Leica DMIL LED inverted microscope (Leica Microsystems), with a HI Plan 10X objective (N.A. 0.22) and equipped with a Leica EC3 digital camera. Three independent experiments were performed and at least 12 random fields were counted per experiment. Datasets were normalized and plotted against HCT116 control cells.

For invasion assay, Matrigel (BD) was diluted with PBS to a final concentration of 3 mg/mL and polymerized in transwell inserts (Corning) at 37 °C for at least 1 h. 10^5^ cells were seeded directly onto the matrigel in 1% FBS medium. Transwell inserts were finally placed in medium supplemented with 10% FBS and cells were allowed to invade at 37 °C for 36 h. Invading cells were fixed and processed as described in transwell migration assay section. Three independent experiments were performed and at least 12 random fields were counted per experiment.

### Immunofluorescence and confocal microscopy

A density of 5 × 10^4^ HCT116 or A549 cells were seeded on eight-well Labteck (Nunc, Roskilde, Denmark) slides pre-coated with 1 mg mL^−1^ collagen (BD Bioscience, Madrid, Spain). Cells were fixed in Saccomanno’s cytology fixative and permeabilized by incubation with 0.5% Triton X-100 at room temperature for 5 min. Non-specific binding was blocked by incubation with 1/10 goat serum (Sigma–Aldrich) for 30 min at room temperature. Incubation with a specific anti-CTNNB1 (CST-9562) antibody and was carried out overnight. Samples were incubated 1 h at room temperature with secondary Alexa fluor 594 goat anti-rabbit IgG (Invitrogen) and DRAQ5^TM^ for nuclear visulaization. For image acquisition, LSM 800 (Zeiss, Jena, Germany) inverted confocal microscope equipped with a 63x Plan-Apochromat objective (NA1.4 oil) was used. Images were acquired using the Zen 2.3 software. All images were captured and processed using Volocity Software (Perkin Elmer, Waltham, MA, USA). Images analyses were performed using ImageJ software (Bethesda, MD, USA).

### Statistical analysis

Normally distributed data were analyzed using a Student’s *t*-test. Data with a non-parametric distribution were analyzed using the Kruskal–Wallis and Mann–Whitney U tests. Differences were considered significant at *P* < 0.05.

### Generation of mutant clones with CRISPR-Cas9

Two sgRNAs were cloned separately into pX300 plasmids [52] and transfected together with a plasmid containing GFP in HCT116. GFP positive cells were sorted and raised individually in M96-plate wells. Positive clones were then identified by PCR using a pair of primers flanking the depleted region.

### Accession numbers

The primary data from the microarray analyses are available at the Gene Expression Omnibus (GSE98928) [53].

### Oligonucleotides

The list of oligonucleotides is in Additional file 1.

## Additional files

Additional file 1: Figure S1. LINC-PINT is downregulated in colon and lung adenocarcinoma. **Figure S2.** LINC-PINT is localized in cell nucleus and LINC-PINT overexpression in HCT116 decreases tumor formation in vivo. **Figure S3.** A highly conserved short region of LINC-PINT is required for its function. **Figure S4.** LINC-PINT inhibits a pro-invasion gene signature. **Figure S5.** LINC-PINT inhibits the expression of EGR1 transcriptional target genes. **Figure S6.** PRC2 mediates the LINC-PINT-dependent silencing of pro-invasion genes. List of oligonucleotides (PDF 4621 kb)

Additional file 2: Table S1. shows the genes affected upon LINC-PINT overexpression. (XLSX 81 kb)

Additional file 3: Table S2. shows the biofunctions associated to genes affected upon LINC-PINT overexpression. (XLSX 109 kb)

## Abbreviations

ASO: Antisense oligonucleotide
CRISPR: Clustered regularly interspaced short palindromic repeats
EZH2: Enhancer of zeste homolog 2
FISH: Fluorescence in situ hybridization
LINC-PINT: Long intergenic non-coding-p53 induced non-coding transcript
lncRNA: Long non-coding RNA
PRC2: Polycomb Repressive Complex 2
qRT-PCR: Quantitative reverse transcription polymerase chain reaction
SUZ12: Suppressor of zeste 12
