# Supplementary material for: The human lncRNA LINC-PINT inhibits tumor cell invasion through a highly conserved sequence element
Source: Genome Biol. 2017 Oct 27;18:202. doi: 10.1186/s13059-017-1331-y (PMC5660458; doi:10.1186/s13059-017-1331-y)
Supplement: Supplementary file 1 — LINC-PINT is downregulated in colon and lung adenocarcinoma. Figure S2. LINC-PINT is localized in cell nucleus and LINC-PINT overexpression in HCT116 decreases tumor formation in vivo. Figure S3. A highly conserved short region of LINC-PINT is required for its function. Figure S4. LINC-PINT inhibits a pro-invasion gene signature. Figure S5. LINC-PINT inhibits the expression of EGR1 transcriptional target genes. Figure S6. PRC2 mediates the LINC-PINT-dependent silencing of pro-invasion genes. List of oligonucleotides (PDF 4621 kb) [file 13059_2017_1331_MOESM1_ESM.pdf]

SUPPLEMENTARY FIGURES

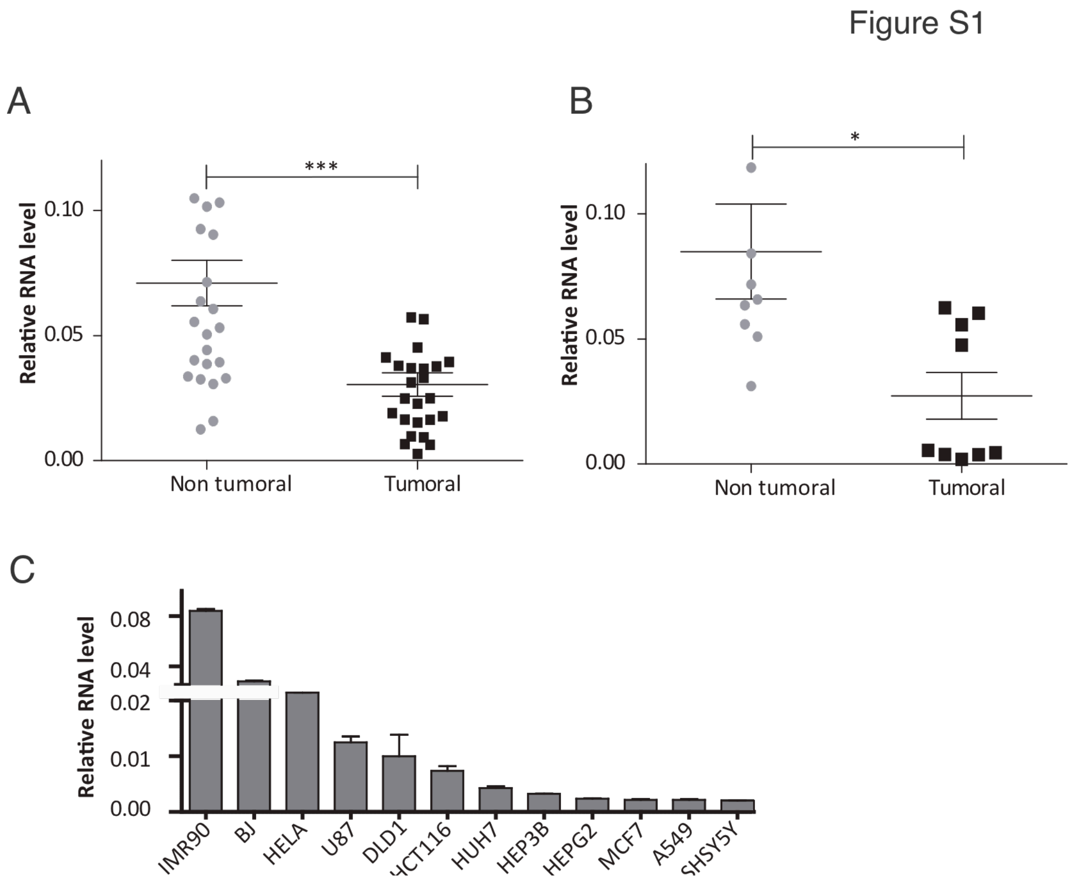

Figure S1. LINC-PINT is downregulated in colon and lung adenocarcinoma.

(A) *LINC-PINT* relative expression level in colorectal tumor samples and non-tumoral peripheral tissue.
(B) *LINC-PINT* relative expression level in lung adenocarcinoma paired patient samples.
(C) *LINC-PINT* relative expression level in cell lines.
Two tails t-test was performed and the statistically different values are indicated (*P<0.05 **P<0.01***P<0.001).

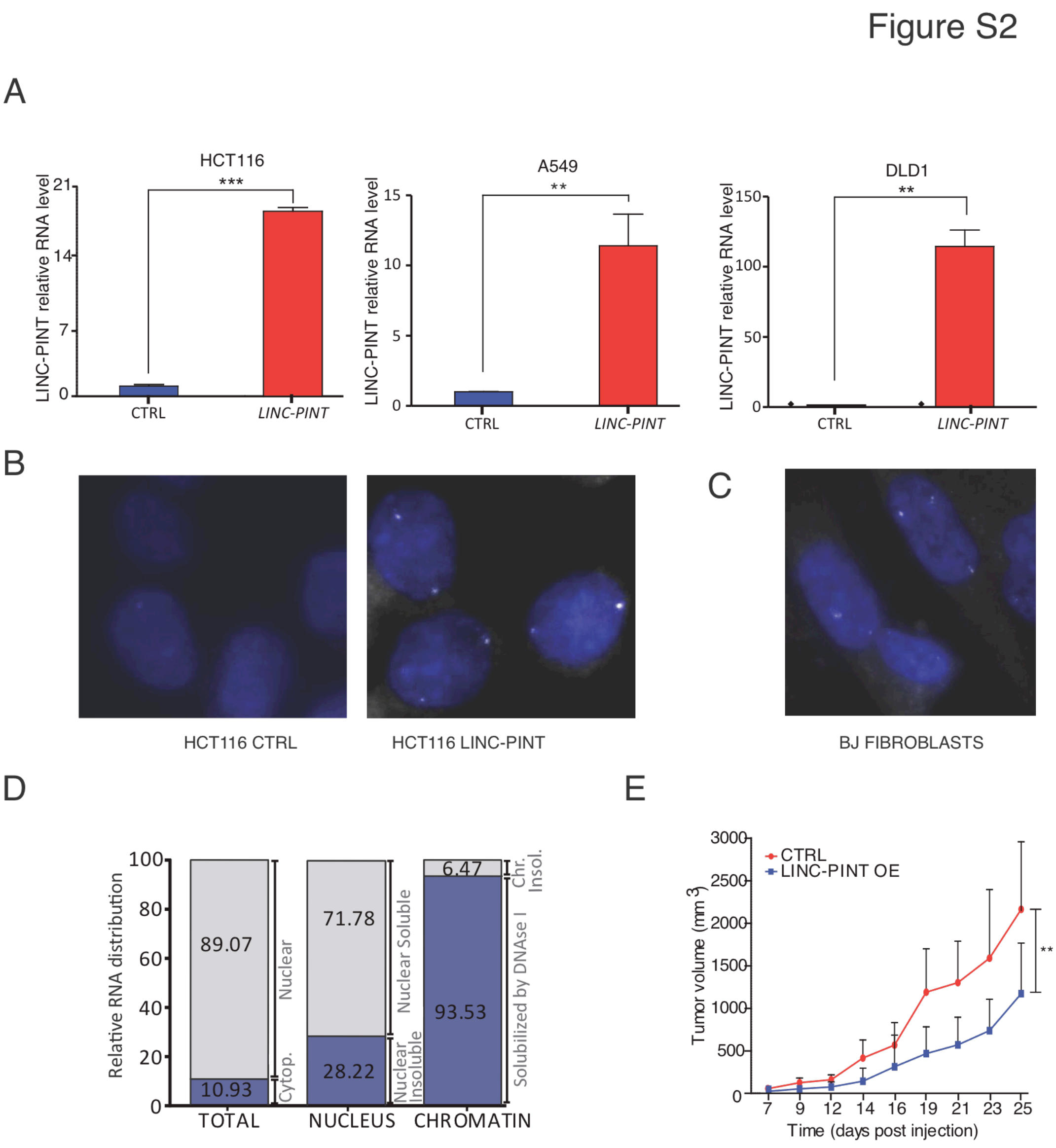

Figure S2. *LINC-PINT* is localized in cell nucleus and *LINC-PINT* overexpression in HCT116 decreases tumor formation *in vivo*.

(A) *LINC-PINT* relative expression level after stable overexpression in HCT116, A549 and DLD1 cancer cell lines.

(B) and (C) Representative *LINC-PINT* single-molecule RNA FISH images of HCT116 control and HCT116 *LINC-PINT* cells (B) and BJ human fibroblasts (C).

(D) Relative *LINC-PINT* distribution in cytoplasmic, nuclear, and chromatin subcellular RNA fractions of human BJ fibroblasts.

(E) HCT116 control and *LINC-PINT* overexpressing cells subcutaneously injected in immunodeficient nude mice (n = 6 per experimental condition). Tumor volume was measured at the indicated times. Graph shows mean ± SD of n = 6 mice per experimental condition. Significance was determined by t-test comparing tumor sizes at final time points (CTRL vs *LINC-PINT*).

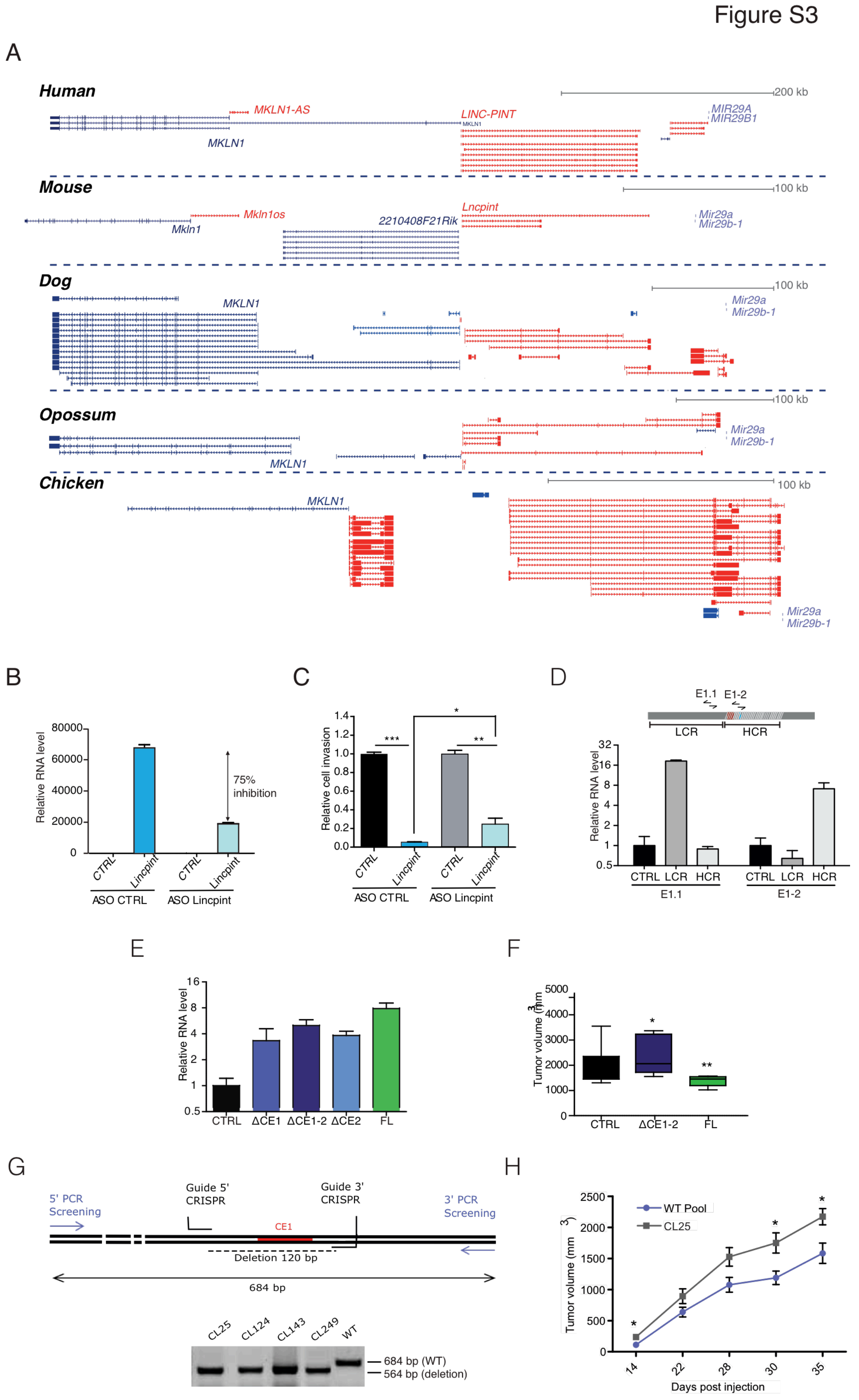

Figure S3. A highly conserved short region of LINC-PINT is required for its function

(A) LINC-PINT genomic locus (region between MKLN1 and MIR29A/B1) is shown in five species. Gene annotations in human and mouse are from RefSeq. MLKN1 and MIR29 annotations in Chicken is from Ensembl. Other annotations are from RNA-seq based transcriptome reconstructions {Hezroni, 2015 #2436}. The colors indicate the strand of transcription (red = left to right; blue = right to left).
(B and C) Relative murine *Lincpint* RNA levels (B) and invasion capacity (C) of control and *Lincpint*-overexpressing HCT116 cells, transfected with ASO control or ASO *Lincpint.*
(D) Relative expression of *LINC-PINT* mutants stably expressed in HCT116. The diagram indicates the location of oligo pairs along the transcript.
(E) Relative level of the full length *LINC-PINT* (FL) or mutants stably expressed in HCT116 cells. Control cells (CTRL) are transduced with an empty vector.
(E) Control, *LINC-PINT,* FL and ΔCE1-2 expressing HCT116 cells are subcutaneously injected in immunodeficient mice (n = 6 per experimental condition). Box-plot graph represents the tumor volume at end point of the experiment (mean ± SD of n = 6 mice per experimental condition). Significance was determined by Mann-Whitney U test (*P<0.05 **P<0.01)
(G) Schematic representation of strategy for deletion of CE1 sequence with CRISPR-Cas9 and PCR screening of the clones obtained.
(H) HCT116 cells from a pool of clones with wild type LINC-PINT (WT Pool*)* or a clone with a deletion in LINC-PINT in CE1 region (CL25) were subcutaneously injected in immunodeficient mice (n = 6 per experimental condition). Box-plot graph represents the tumor volume at end point of the experiment (mean ± SD of n = 6 mice per experimental condition). Significance was determined by Mann-Whitney U test (*P<0.05 **P<0.01)

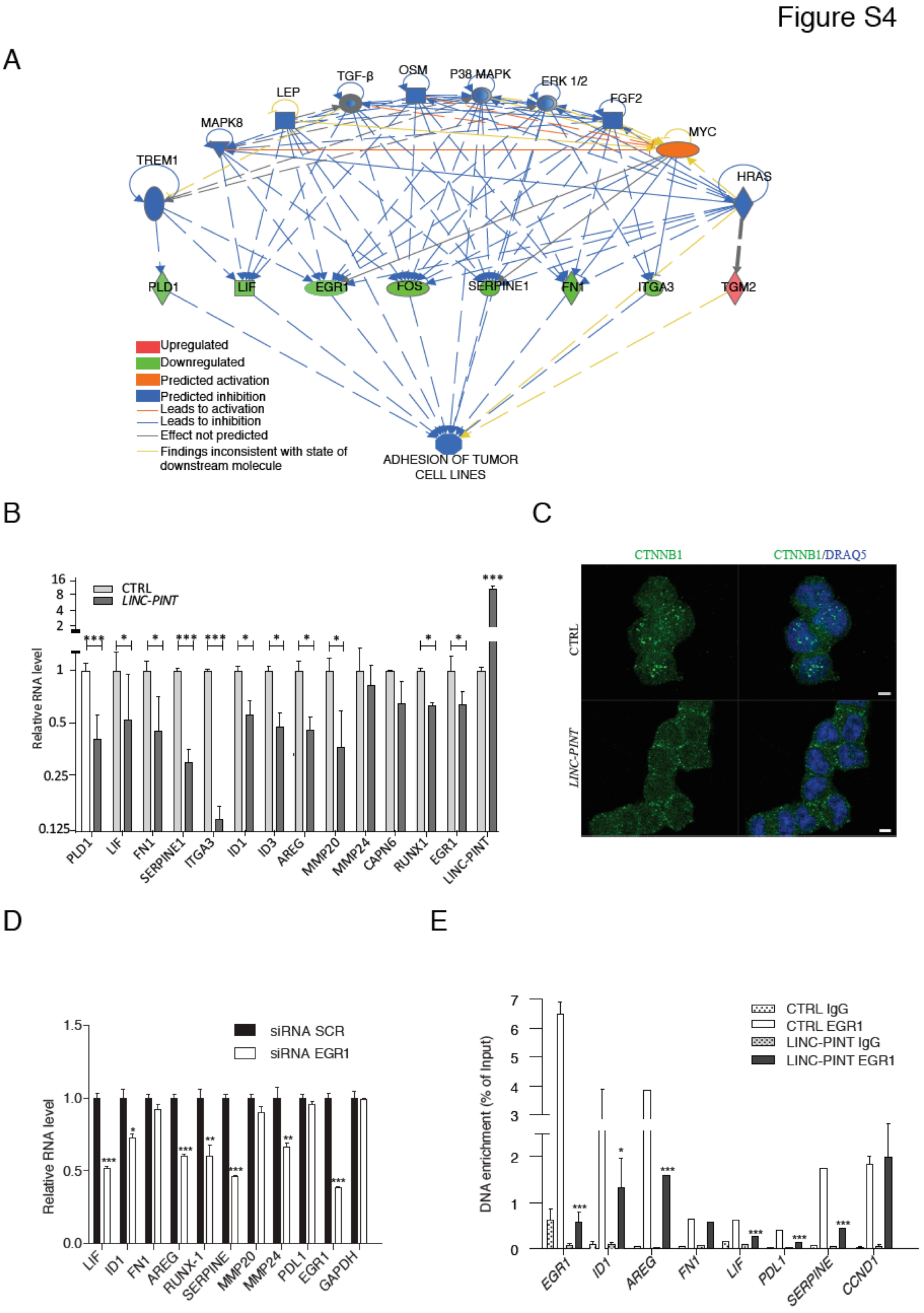

Figure S4. ***LINC-PINT* inhibits a pro-invasion gene signature.**
(A) Network connecting genes differentially expressed in *LINC-PINT* HCT116 cells involved in the regulation of cell adhesion of tumor cell lines as predicted by Ingenuity Pathway Analysis.

(B) Validation of microarray result of HCT116 CTRL and *LINC-PINT* overexpressing cells. Relative RNA expression level of *LINC-PINT* target genes was quantified by qRT-PCR in three independent experiments.
(C) Immunoflorescence images of CTNNB1 (green) and DRAQ5 (blue, nuclear specific marker) in control cells (CTRL) and LINC-PINT overexpressing A549 cells (LINC-PINT) Scale bar: 20µm

Supplementary Figure 5

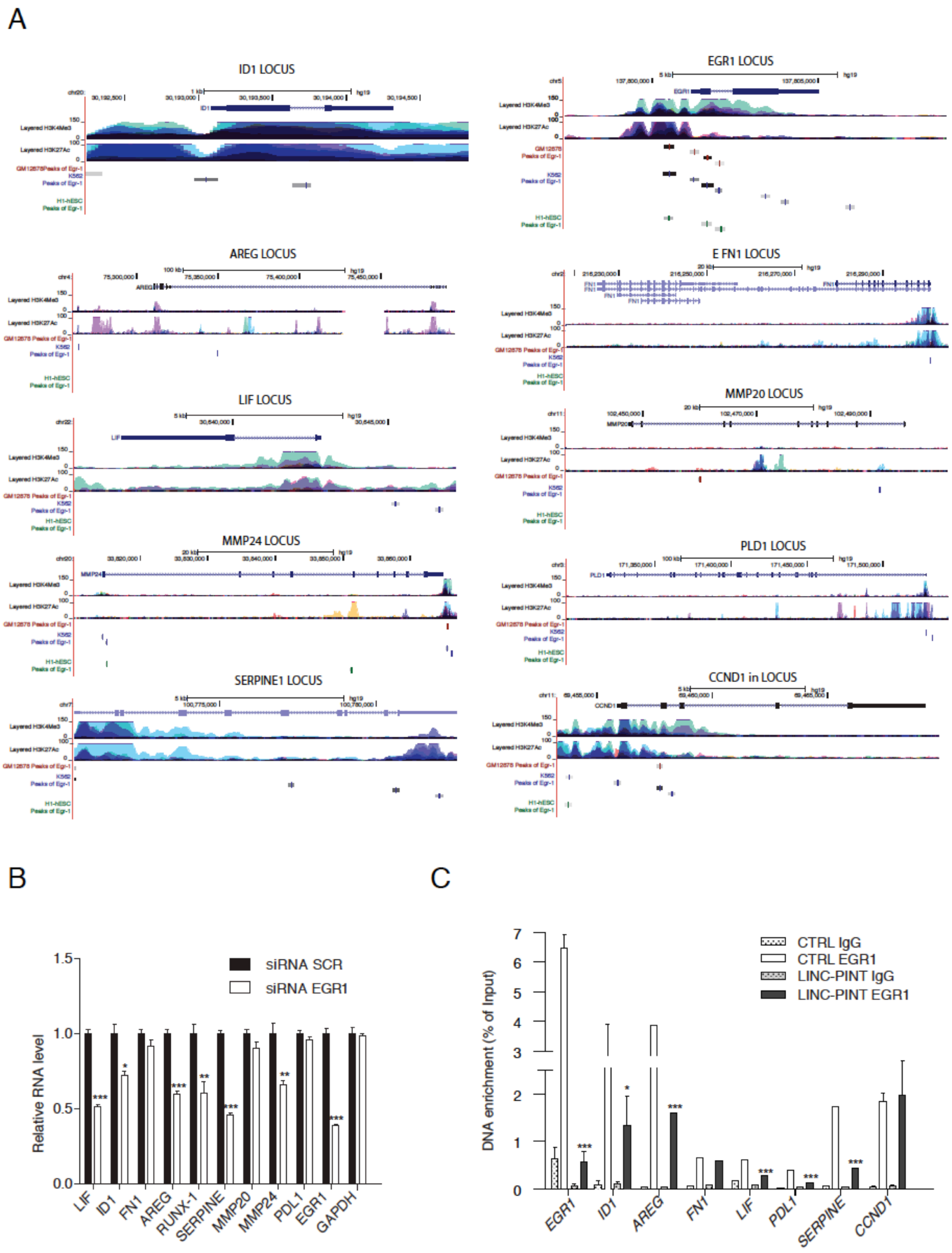

Figure S5. LINC-PINT inhibits the expression of EGR1 transcriptional target genes.
(A) H3K4me3, H3K27Ac ChIP-seq signal, and EGR-1 ChIP-seq peaks in several *LINC-PINT* target gene loci in B GM12878, K562 and H1-hESC. Data are downloaded from https://www.encodeproject.org.
(B) RNA levels of EGR1 gene targets in HCT116 cells treated with non-targeting siRNA control (siRNA SCR) or EGR1 siRNA. Genes are selected based on public EGR1

ChIP-seq data and their downregulation in LINC-PINT cells. GAPDH is shown as and unrelated control gene. The RNA levels are calculated relative to HPRT, and the statistic significance of differences between siRNA SCR and siRNA EGR1 is indicated with the stars.
(C) EGR1 (and unspecific IgG as control) ChIP-qPCR of genes that are EGR1 targets and downregulated by LINC-PINT in control (CTRL) or LINC-PINT overexpressing HCT116 cells (LINC-PINT). CCND1 is shown as control, as it is EGR1 target but not regulated by LINC-PINT. Statistical differences between CTRL and LINC-PINT cells in EGR1 ChIP samples are indicated.
Significance was determined by two-tailed unpaired t-test and the statistically different values are indicated (*P<0.05 **P<0.01***P<0.001).

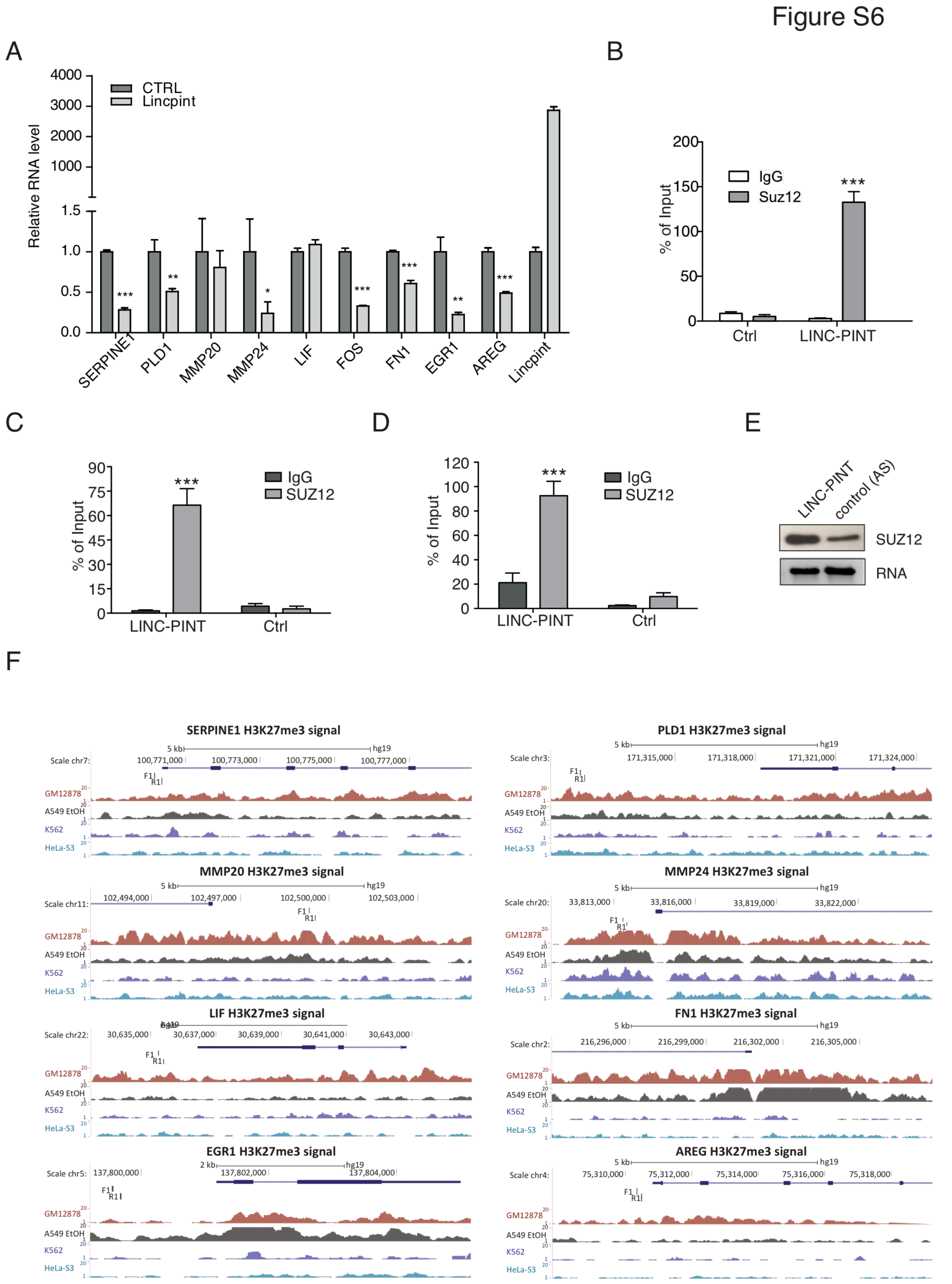

G

Position	Length	QGRS	Score
3	29	GGAGAGGAAAGCAUUAGGGGAGCCCACGG	15

Figure S6. PRC2 mediates the LINC-PINT-dependent silencing of pro-invasion genes

(A) Relative expression level of pro-invasion genes in HCT116 cells with overexpression of the murine *Lincpint*. Significance was determined by two-tailed unpaired t-test and the statistically different values are indicated (*P<0.05 **P<0.01***P<0.001).
(B) RNA Immunoprecipitation of Suz12 in A549 cells followed by qRT-PCR of *LINC-PINT* and ATM (Control -).
(C) RNA Immunoprecipitation of Suz12 in A549 cells crosslinked with 0.5% formaldehyde followed by qRT-PCR of *LINC-PINT* and ATM (Control -).
(D) RNA Immunoprecipitation of Suz12 in HCT116 crosslinked cells with UV followed by qRT-PCR of *LINC-PINT* and GAPDH (Control -).
(E) Detection of SUZ12 protein enriched in RNA pulldown experiment with *in vitro* synthesized LINC-PINT or antisense LINC-PINT (control) incubated with purified PRC2.
(F) H3K27me3 ChIP-seq signal in several *LINC-PINT* target gene loci in B-Lymphocyte-derived cell line GM12878 and A549, K562 and HeLa cancer cell lines. Images are downloaded from UCSC genome browser.
(G) Prediction of G-quadruplexes within CE1 domain of LINC-PINT identified by *QGRS-H predictor* {Kikin, 2006 #3031}.

SUPPLEMENTARY TABLE LEGENDS

Table S1. Genes with expression changes (P<0.01 in HCT116 LINC-PINT compared to HCT116 control cells.

Table S2. Biofunctions enriched in the genes differentially expressed in HCT116 LINC-PINT cells.

OLIGONUCLEOTIDES

qRT-PCR

PLD1.F	AGCTCCAGCTGTGCCAGA
PLD1.R	TTTGGACAGAGTAAAAGCAAAGG
LIF.F	TGCCAATGCCCTCTTTATTC
LIF.R	ACCAGCTTGGCCTTCTCC
FN1.F	GCGAGAGTGCCCCTACTACA
FN1.R	GTTGGTGAATCGCAGGTCA
SERPINE1.F	CCAGCTGACAACAGGAGGAG
SERPINE1.R	CCCATGAGCTCCTTGTACAGAT
ITGA3.F	AACCAGGATGGATTTCAGGA
ITGA3.R	CAGTCCCAGCTTCTCTCCAT
ID1.F	CCAGAACCGCAAGGTGAG
ID1.R	GGTCCCTGATGTAGTCGATGA
ID3.F	CATCTCCAACGACAAAAGGAG
ID3.R	CTTCCGGCAGGAGAGGTT
AREG.F	TGATCCTCACAGCTGTTGCT

AREG.R	TCCATTCTCTTGTCGAAGTTTCT
MMP20.F	CCCCCTCCCTAGTTGCAG
MMP20.R	TGTAATATTTGTCAAGATACGCCTGT
MMP24.F	TCTCCAGGGCATCCAGAA
MMP24.R	GAGTGTAGGGAGTGGCCTTG
CAPN6.F	TTGTCCCAACCATGTTCCA
CAPN6.R	GGGCATGTCCAGAGTCAGTT
RUNX1_F	ATGAGGGTCAGCCCACAC
RUNX1_R	TGGTTGGATCTGCCTTGTATC
EGR1_F	CTTCAACCCTCAGGCGGACA
EGR1_R	GGAAAAGCGGCCAGTATAGGT
mLincpint.F	CGGTGTAGTGTTCAGCCTCA
mLincpint.R	GGTGGCAGACTCCTGTTAGC
Ezh2.F	GCTGACCATTGGGACAGTAA
Ezh2.R	CAGATGGTGCCAGCAATAGA
hPINTex1.0.FWD	TTCTGTTTTCCCAGCGATCT
hPINTex1.0.REV	ACAGAAGGAGCGTCCTCAAA
hPINTex1.1.FWD	GGAAGAGAAACTACGCCACCT
hPINTex1.1.REV	GCCGGCTAAAAGTTGTCCT
hPINT ex1.2F	GTCATGAGCACAGGCTCCAC
hPINT ex1.2R	ACAACAGCAGCAGGACTGG
PINT_intron1.0.F	AGCTACTCGGGAGGGCTAAG
PINT_intron1.0.R	ACGGTGTTGTTCTGTCACCA
PINT_intron1.1.F	GAGACCGTCCTGGCTAACAC
PINT_intron1.1.R	CATGCCATTCTCCTACGTCA
hPINTex2.FWD	CGCGGAGGACAACTTTTAG
hPINTex2.REV	CTTGCCTCGTTCCTTCCTC
PINT_intron2.0.F	TAGGGATTTTGGGGGAAGAC
PINT_intron2.0.R	AGCTAAGCGAATGGAGTGGA
hPINTex2-3.FWD	GAACGAGGCAAGGAGCTAAA
hPINTex2-3.REV	AGCAAGGCAGAGAAACTCCA
PINT_intron3.0.F	AGATGTGGTTCCTGGTGAGG
PINT_intron3.0.R	CCCCATATTGGGATTTGTGT
PINT_ex4F	GCTGAGCCTCCTACCTCATCT
PINT_ex4R	AAGGAACGAGCCAGGAAGAG
PINT_CE1_F	CCTCTCTGGTGTCACATGAT
CTNNB1 Fwd	GCCTTCGTTCTCTTCCCTTT
CTNNB1 Rev	CATTCACCACTCACAAACTCG
CDKN1A Fwd	CCGAAGTCAGTTCCTTGTGG
CDKN1A Rev	CATGGGTTCTGACGGACAT

ChIP qPCR

hGAPDHprom.F	AGTTGGAATTCACACCCATGA
hGAPDHprom.R	GGTCAAGGCTGCAATTAGTCA
FN1.CHIP.F	GGGGAGAGAAAGGTCTTCGT

FN1.CHIP.R	CGAAGGAGGGCAAGACAGTA
PLD1.chip.F	CAGAGTGGGAATACTGGGTCA
PLD1.chip.R	GTGGCCACAGGTAAACTCAGA
LIF.chip.F	GGCACAGGAGCTGACACTTAC
LIF.chip.R	ACTGGGAAACCACAGACACTG
Serpine1.chip.F1	GGTTTGCTCAATTGTTCCTGA
Serpine1.chip.R1	GCCACTGCCTCCTTTTATACC
AREG.chip.F	ATAGGGAGTGAGGGGATTTGA
AREG.chip.R	ACCAAGAGTGACTTTGCCTGA
MMP20.chip.F	GGCTGGTGACGATGTTTCTTA
MMP20.chip.R	GGCCATGGTAAGTCTGGGTAT
MMP24.chip.F	CCCATTTTCCTTCAGAACACA
MMP24.chip.R	AGGGAGGAAGTTGAGGGTGTA
EGR1_CHIP.F	CGGTCCTGCCATATTAGGGC
EGR1_CHIP.R	CCCGGATCCGCCTCTATTT

CRISPR Cas9

5' guide	GCCTCTCTGGTGTCACATGA
5' guide (RC)	TCATGTGACACCAGAGAGGC
3' guide	GCTGCGTGCACTCGGCTGCA
3' guide (RC)	TGCAGCCGAGTGCACGCAGC
5' Screening	TTCTGTTTTCCCAGCGATCT
3' Screening	GCCGGCTAAAAGTTGTCCT

shRNA

shEZH2.F	CCGGCGGCTCCTCTAACCATGTTTACT
shEZH2.R	AATTCAAAAACGGCTCCTCTAACCATG
shscramble.F	CCGGCAACAGCCACAACGTCTATATCT
shscramble.R	AATTCAAAAACAACAGCCACAACGTCT

ASOs

CTRL	Ionis ID 141973
h7	CAGAGTATGTACTTCTCAAC
h8	GTGGATGCTTTGTTTCTCAA

siRNAs

EGR1	GGACAAGAAAGCAGACAAA
SCR	CAGUCGCGUUUGCGACUGG
